# Serum circulating miRNA‐342‐3p as a potential diagnostic biomarker in parathyroid carcinomas: A pilot study

**DOI:** 10.1002/edm2.284

**Published:** 2021-07-29

**Authors:** Julia Krupinova, Natalya Mokrysheva, Vasiliy Petrov, Ekaterina Pigarova, Anna Eremkina, Ekaterina Dobreva, Alina Ajnetdinova, Galina Melnichenko, Anatoly Tiulpakov

**Affiliations:** ^1^ Endocrinology Research Center Moscow Russia

**Keywords:** microRNAs, parathyroid carcinoma, parathyroid gland

## Abstract

**Objective:**

To compare the serum miRNA expression profiles between patients with benign and malignant parathyroid tumours.

**Background:**

Despite recent advances in molecular biology, a histological tissue biopsy is still the method of choice used to diagnose most cancers. The preoperative cytology is not an applicable method for diagnosis of parathyroid cancer (PC); therefore, huge interest exists in terms of finding alternative methodologies to seek specific cancer biomarkers.

**Design:**

A retrospective cross‐sectional study.

**Patients and Methods:**

Serum samples of patients with PC (*n* = 13) and parathyroid adenoma (PA) (*n* = 11), age (*p* = .999) and sex (*p* = .999) were matched and examined via the simultaneous comparative expression analysis of 754 microRNAs (miRNAs). The «TaqMan OpenArray Human MicroRNA Panel» (Applied Biosystems) was used to conduct real‐time PCRs using the «QuantStudio 12К Flex» station (Life Technologies).

**Results:**

According to the results of a pilot study, significant changes in expression levels between the PC group and the PA group (control) (*p* < .05) were observed for 17 miRNAs. Among them, the downregulation of miRNA‐342‐3p met the Benjamini‐Hochberg adjustment criteria for multiple comparisons (*p *= .02).

**Conclusions:**

Serum miRNA‐342‐3p could be a promising biomarker for PC to improve diagnosis and prognosis.

## Graphical abstract

The main objective of this study was to compare the serum miRNA expression profiles between benign and malignant parathyroid tumors. To the best of our knowledge, this study is the first report on serum miRNA profiles derived from PC patients.

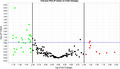

## 1 INTRODUCTION

Parathyroid carcinoma (PC) is a rare and aggressive malignancy, accounting for less than 1% of primary hyperparathyroidism (PHPT) and only 0.005% of all cancers.^1^, ^2^ Five‐year survival rates, as pooled from different registries and case series, are in the range of 76%–85%, with 10‐year survival rates being between 49% and 77%.^3^, ^4^ Recurrence occurs in more than half of PC cases, with the five‐year survival rate in patients with metastatic disease being less than 50%.^5^, ^6^ Reports from the United States, Australia, Finland and the Russian Federation indicate an increasing incidence of PC,^2^, ^7^, ^8^ which may be related to improvement in diagnostics or to an actual increase in incidence. The PC pathogenesis is currently unclear, but it is known that the cell division cycle 73 (*CDC73)* germline inactivation mutations can play important roles. *CDC73*, which is comprised of 17 exons and is located on chromosome 1q31.2, encodes the protein, parafibromin, which is associated in the polymerase‐associated factor (Paf1) complex. Functions attributed to parafibromin include the downregulation of cyclin D1 expression and direct interaction with β‐catenin resulting in the activation of transcription of target genes. More than 50% of *CDC73* germline mutations associated with hyperparathyroidism‐jaw tumour syndrome (HPT‐JT), as well as about 15% of *CDC73* germline mutations, were reported in patients with suspected sporadic PC.^9^

The prognosis of PC is greatly influenced by surgeon performance, which emphasizes the importance of preoperative diagnosis.^10^ In contrast to benign parathyroid tumours, which are effectively treated by selective parathyroidectomy, PCs require *en bloc* resection implying that this avoids capsule rupture. An insufficient volume and experience of the surgeon increase the risk of distant metastasis, the treatment of which has extremely limited efficiency.^11^ Achieving microscopic, cancer‐free margins improves disease‐free survival.^12^ Nevertheless, only 12.5% of patients with PC undergo radical surgery.^7^

Despite recent advantages in molecular biology, a histological tissue biopsy is still the method of choice to diagnose most cancers, with the exception of PC. Cytology biopsies of the parathyroid gland were reported to be uninformative, even dangerous due to the possible diffusion of malignant cell seeding along the needle track.^13^ Therefore, the majority of PC cases are diagnosed postoperatively by histological examination,^11^ presenting an important opportunity to identify new preoperative PC markers to aid in the success of initial operations, thus improving the disease‐free survival of patients.

Increasing evidence suggests that microRNAs (miRNAs) may be a new group of biomarkers for various cancers. MiRNAs are small, endogenous, noncoding RNAs about 23 nucleotides long. To date, around 2500 miRNA sequences have been identified in humans (miRBase database 20.0).^14^ In theory, one miRNA can target hundreds of genes and one gene can be targeted by multiple miRNAs. They are involved in almost all biological processes, such as cell proliferation, apoptosis and tumorigenesis, and play a crucial biological role in tumorigenesis and progression, as they affect cell proliferation, differentiation, adhesion, migration, invasion and apoptosis.^15^ Moreover, alterations in miRNA expression have been observed in many diseases, including various types of cancer, indicating that miRNAs might be useful for cancer management. Unlike long molecules of RNA (eg mRNA), circulating miRNAs are highly stable in the majority of fluids. In recent years, much attention has been paid to the detection of such biomarkers in body fluids.^16^ An important feature of miRNAs is their ability to circulate within the blood, which makes it possible to use them as high‐quality biomarkers of human cancer.^17^, ^18^ Several studies analysed miRNA expression in parathyroid tissue, including PC,^19^, ^20^, ^21^, ^22^ but there is a lack of research investigating circulating miRNA levels in PC patients. The main objective of this study was to compare the serum miRNA expression profiles between benign and malignant parathyroid tumours. To the best of our knowledge, this study is the first report on serum miRNA profiles derived from PC patients.

## 2 MATERIALS AND METHODS

### 2.1 Patients and sample collection

All patients gave their informed consent for inclusion before they participated in the study. The study was conducted in accordance with the Declaration of Helsinki, and the Ethics Committee of the Endocrinology Research Centre approved the protocol (Moscow, Russia; protocol #1 of 01/25/2017).

Fasting blood samples were collected from the patients with biochemically confirmed active PHPT, centrifuged within two hours of collection at 3500 rpm for 15 min at +4°C. Subsequently, serum was aliquoted and stored at −80°C until further use. We included 13 patients with PC and 11 patients with PA after retrospective analysis based on morphological diagnosis (results confirmed by two independent morphologists) according to inclusion and exclusion criteria. Histopathological diagnosis of parathyroid tumours was established according to the WHO classification criteria.^23^ Exclusion criteria comprised of accompanying terminal chronic kidney disease, malignant neoplasms of other organs, severe comorbidities (heart failure stage III and IV by the New York Heart Association (NYHA)), diabetes mellitus type 1 or 2, mental illness, acute respiratory viral infections, exacerbation of chronic disease during the last month, pregnancy and lactation, and intake of calcium‐phosphorus–related medications. Biochemical parameters of fasting blood (serum total calcium (reference interval (RI) 2.15–2.55 mmol/L), ionized calcium (RI 1.03–1.29 mmol/L), and creatinine (RI 63–110 mcmol/L)) were determined using the automatic biochemical analyser ARCHITECT c8000 (Abbott). Intact parathyroid hormone (iPTH, RI 15–65 pg/mL) was evaluated using the second‐generation electro‐chemiluminescent analyser Cobas 6000 (Roche).

### 2.2 MiRNA isolation from serum

The miRNAs were isolated from the serum samples using the mirVana PARISTM kit (Life Technologies) as per the total RNA isolation procedure for liquid samples according to the manufacturer's recommendations. Briefly, 300 µl of each serum was mixed with 300 µl of 2 × denaturing solution at room temperature, combined with 600 µl of acid‐phenol: chloroform and mixed thoroughly with vortex. Then, the sample lysate was centrifuged at 15,000 g for 5 min to separate the mixture into aqueous and organic phases. The aqueous phase was transferred to a fresh tube, mixed with 750 µl of 96% ethanol and applied to a filter cartridge. The filter cartridge was washed once with 700 µl of miRNA wash solution and twice with 500 µl of wash solution 2/3. The RNA was eluted from the filter in 100 µl of preheated (95°C) elution solution and stored at −70°C.

### 2.3 Reverse transcription of RNA with Megaplex ^TM^ Trademark RT Primers and pre‐amplification of RT products

Megaplex Trademark (TM) reverse transcription (RT) Primers and Human Pools A and B v3.0 (PN 4444282) were used according to their matching TaqMan Open Array Human miRNA Panel Quant StudioTM 12K Flex. Each pool contained reverse transcription primers for 377 unique miRNAs; the miRNA targets represented in Pool A tended to be better characterized, more broadly expressed, and/or expressed at the higher levels compared with Human Pool B. Each RNA sample was reverse‐transcribed separately with Pools A and B, according to QuantStudio™ 12K Flex OpenArray microRNA Starter Kit protocol (Life Technologies, Publication Part Number 4470935 Rev. C, April 2014). A total of 3 µl of isolated miRNA samples were reverse‐transcribed using the Megaplex. The content of miRNA fractions in the indicated RNA is very small (not detectable); therefore, the protocol recommends using a fixed volume (3 µl) of the isolated RNA in the reverse transcription reaction.

RT Primer Pool A or B stayed in a final volume of 7.5 µl. The RT reaction was performed in a thermal cycler (40 cycles: 2 min at 16°C, 1 min at 42°C, 1 s at 50°C and 5 min at 85°C for enzyme inactivation).

A total of 2.5 µl of each RT reaction was combined with 12.5 µl of 2xTaqMan PreAmp Master Mix (PN 4391128), with 2.5 µl of its corresponding Megaplex PreAmp Primer Pool A or B (PN4444304) and 7.5 µl of nuclease‐free water in a final volume of 25 µl. A pre‐amplification reaction was run under the following conditions: (1) hold for 10 min at 95°C, 2 min at 55°C and 2 min at 72°C; (2) perform 12 cycles: 15 sec at 95°C, 4 min at 60°C; (3) hold for 10 min at 99°C; and (4) keep at 4°C. A total of 4 µl of each pre‐amplification product, corresponding to the RNA samples of three patients, was diluted to 1:40 with a low TE buffer (Low Tris/EDTA buffer) =10mM TrisCl (8.0), 0.1mM EDTA for RT PCR. Other pre‐amplified products were stored at −20°C for up to 1 week.

### 2.4 Real‐time PCR on QuantStudio™ 12K Flex

To prepare PCR mixes A and B, 22.5 µl of each diluted pre‐amplification product was combined with 22.5 µl of the 2x TaqMan OpenArray Real‐Time PCR Master Mix (PN 4462159). A total of 5 µl of each PCR mix was transferred to the OpenArray 384‐well sample plate in the combination as described in the microRNA Starter Kit protocol mentioned above. The QuantStudio OpenArray AccuFill™ System was used to transfer the reaction mixes from the 384‐well sample plate to the QuantStudio™ 12K Flex TaqMan OpenArray plate, as described in the protocol mentioned above. The loaded OpenArray plate was run on a QuantStudio™ 12K Flex System under the preprogrammed cycling conditions that applied to the OpenArray experiments.

### 2.5 DNA sequencing

Genomic DNA was extracted from peripheral leukocytes using PureLink1Genomic DNA Mini Kits (Thermo Scientific). A custom SeqCap EZ Prime Choice panel (Roche Sequencing Solutions) targeting 22 genes (*AIP*, *AP2S1*, *CDC73*, *CDKN1A*, *CDKN1B*, *CDKN1C*, *CDKN2A*, *CDKN2C*, *CDKN2D*, *DICER1*, *GATA3*, *GCM2*, *GNA11*, *GNAS*, *MEN1*, *PRKAR1A*, *PRKCA*, *PTTG2*, *SDHA*, *SDHB*, *SDHC*, *SDHD*) was used for the preparation of the DNA library using NimbleGen (SeqCap EZ Prime Choice Library) technology (Roche Sequencing Solutions). Sequencing was performed using an Illumina MiSeq sequencer (Illumina).

### 2.6 Statistical analysis

Statistical analysis was performed using the software packages STATISTICA 13 (StatSoft) and SPSS (IBM). The quantitative results were reported as the median and the 1st and 3rd quartiles [Q1; Q3]. Spearman's rank test was conducted to assess the correlation between miRNA and laboratory indicators. A ROC curve was constructed, and the AUC was calculated to evaluate the diagnostic value of the selected miRNA and it combination with serum calcium and iPTH levels. The cut‐off point was selected so that the sum of the diagnostic sensitivity and diagnostic specificity was the maximum for it. Differences in the expression of miRNA and age between groups were evaluated using the Mann‐Whitney test (U test) as well as differences in terms of quantitative characteristics. The frequencies of the signs were compared with each other using the chi‐square test (*χ* ^2^). The Yates amendment was applied when it was necessary. A *p*‐value less than 0.05 was considered statistically significant. For multiple comparisons, the Bonferroni correction and Benjamini‐Hochberg adjustment were applied. The confidence intervals of the frequencies are calculated by the Klopper Pearson method.

### 2.7 Quantification of miRNA

Raw data files (.eds) were analysed using the ExpressionSuite v1.1 data analysis software (Life Technologies). Ct values were normalized using a global normalization algorithm as an option of the ExpressionSuite v1.1 which uses the mean expression value of all expressed miRNAs in each sample as a normalization factor for miRNA real‐time quantitative PCR data.^24^ Targets with amplification scores of <1 and with Cq confidence values of <0.8 were rejected during analysis. For comparison of the miRNA‐level delta cycle relative threshold (ΔCRT) between the PC and PA samples, the differences were considered to be significant when *p*‐values were less than 0.05 after Benjamini‐Hochberg adjustment.^25^ Note, that in data modelling with ExpressionSuite, the Benjamini‐Hochberg algorithm was used as one of the options and the differences in the fold change of log2 <−1 or >1 were considered relevant.

## 3 RESULTS

### 3.1 Clinical and laboratory characteristics of the study population

Twenty‐four patients with PHPT were included in this study (8 males and 16 females). The mean age of patients in the PC group (*n* = 13) was 58 years [28; 63] and 55 years [32; 68] in the parathyroid adenoma (PA) group (the control group, *n* = 11). These groups were age [*p* = .99 (*U* test)] and sex [*p *= .99 (χ2 with Yates correction)] matched.

The median level of iPTH in the PC group was 4.7 times higher than in the control group, 988 pg/ml [543; 1289] vs 211 pg/ml [80; 520], respectively (*p *< .01, *U* test). The median serum calcium level for the PC group was 3.5 mmol/L [3.17; 4.19] and 2.82 mmol/L [2.68; 3.05] in the PA group (*p* < .01, U test). Metastases were observed in eight participants at the diagnosis or during the follow‐up. Germline mutations in the *CDC73* gene were identified in three patients with PC (2% (95% CI, 5%‐54%)) and all of them developed metastases. The clinical suspicion of HPT‐JT syndrome could be made in only one patient who has a familial history of PA and none of the patients had characteristic manifestations of this syndrome. The clinical, biochemical, histological, and genetic characteristics of the patients are listed in Table 1.

**TABLE 1:** Clinical, biochemical, histological and genetic characteristics of the patients

Case	Age at time of diagnosis	Sex	Diagnosis	iPTH (pg/mL)	Calcium (mmol/L)	Ionized calcium (mmol/L)	Metastases	CDC73 mutation
1	13	F	PC	209	2.92	1.39	Neck lymph nodes	c.78delC:p.I26fs; P
2	23	F	PA	410	2.82	1.34	No	n/a
3	24	F	PC	569	4.55	2.03	Neck lymph nodes, lung and liver	c.355C>T:p.Q119X; P
4	25	M	PC	988	4.7	1.5	No	No
5	28	F	PC	249	4.19	2.4	Lungs	c.496C>T p. Gln166; P
6	28	M	PA	1224	3.38	1.82	No	n/a
7	32	F	PA	316	2.82	1.4	No	n/a
8	32	F	PA	78	2.8	1.45	No	n/a
9	39	F	PC	490	3.55	1.6	Neck lymph nodes	No
10	40	F	PC	2 785	3.5	1.62	No	No
11	41	F	PA	78	2.52	1.28	No	n/a
12	55	M	PA	208	3.05	1.44	No	n/a
13	58	F	PC	885	3.5	1.65	Neck lymph nodes and lungs	No
14	58	F	PA	118	2.68	1.12	No	n/a
15	59	M	PC	2 146	4.73	1.87	No	No
16	59	M	PA	625	3	1.54	No	n/a
17	61	M	PC	1 149	2.59	1.18	No	No
18	63	F	PC	1 289	2.89	1.62	Neck lymph nodes, lungs, liver, L1	No
19	68	F	PA	520	3.31	1.68	No	n/a
20	69	M	PA	211	2.8	1.6	No	n/a
21	70	M	PC	543	3.24	1.45	Th 6	No
22	72	F	PC	1 081	3.4	1.48	No	No
23	74	F	PC	2 371	3.17	1.48	Lungs	No
24	79	F	PA	80	2.67	1.3	No	n/a

Abbreviations: F, female; M, male; n/a, not available; P, pathogenic; PA, parathyroid adenoma; PC, parathyroid carcinoma.

### 3.2 Differential expression of serum miRNAs between PC and PA groups

**FIGURE 1:**
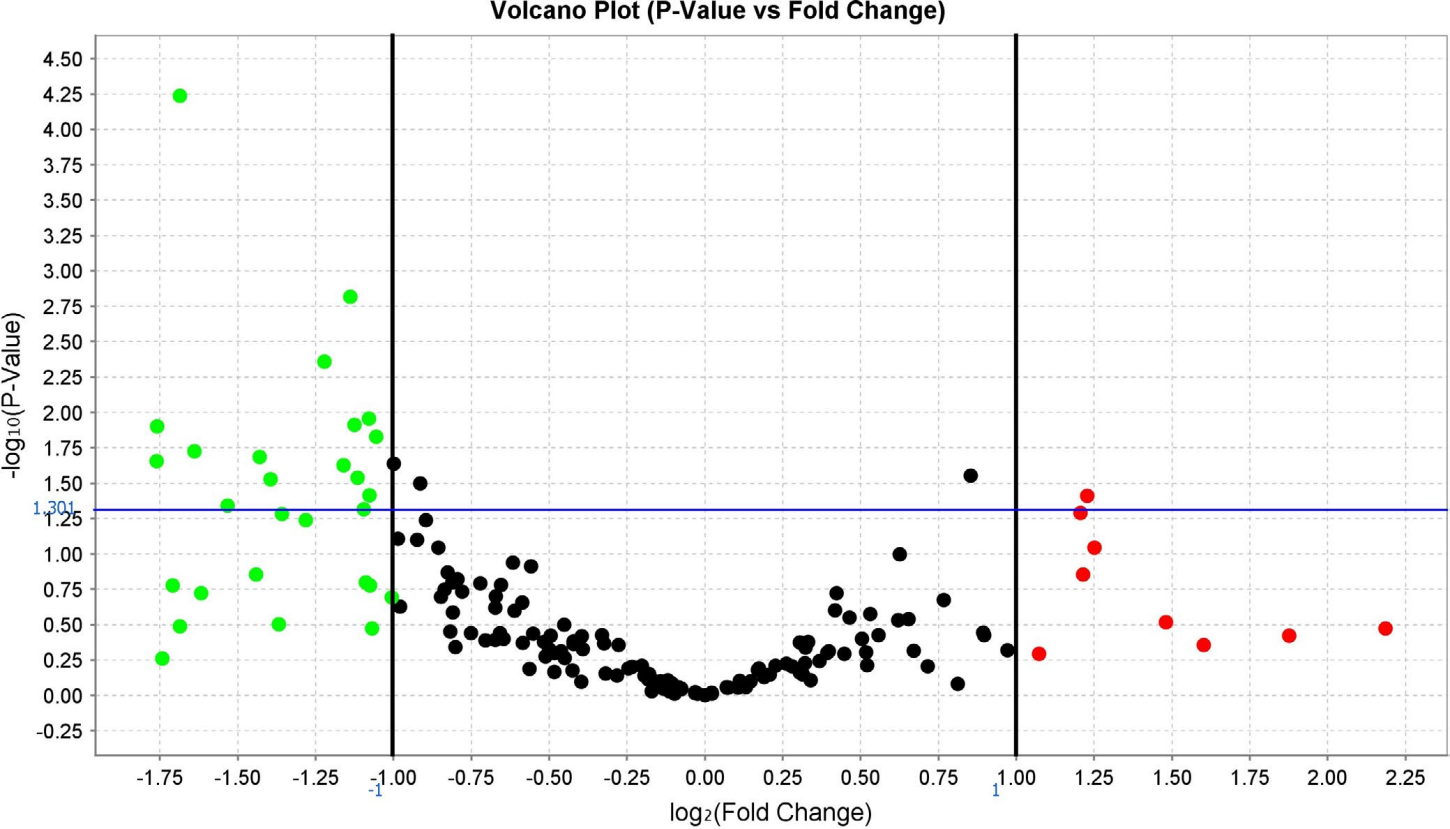
Fold difference in the expression of serum miRNAs (volcano plot) in PC vs. PA patients (control). The fold difference boundary we used in this study is 2.0; green dots are miRNAs with fold change <0.5, and red dots are miRNAs with fold change >2.0. Black dots are miRNAs with not statistically significant. *p*‐value: .05. The blue line (*y*‐axis) corresponds to cut‐off *p*‐value = .05 (‐lg (0.05) =1.301)

**FIGURE 2:**
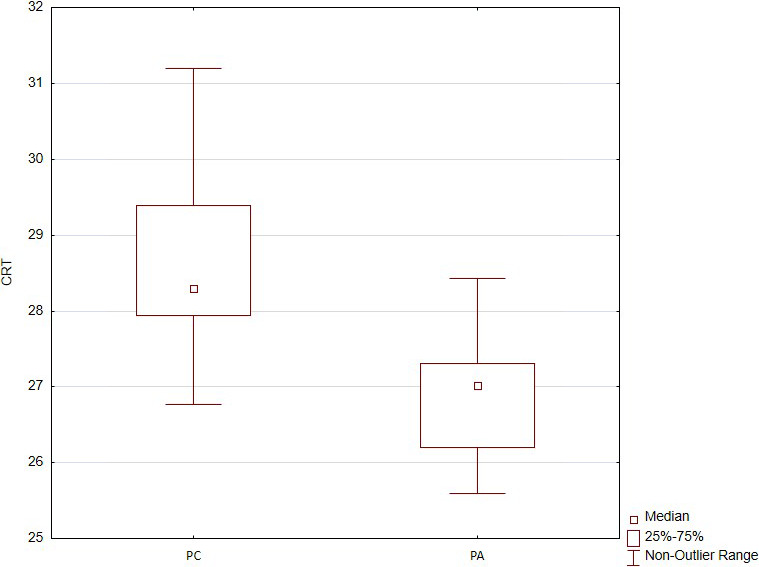
Expression of the serum cycle relative threshold (CRT) miRNA‐342‐3p in the PC group and the PA group (control)

From a simultaneous comparative analysis of 754 serum miRNA types, significantly different expression levels between the PC group and the control PA group (*p* < .05, *U* test) were detected in 17 miRNAs (Supplementary). miRNA‐342‐3p is significantly different, and a further 16 miRNAs demonstrated a *p*‐value of <.05 (Figure 1). Among these levels, after Benjamini‐Hochberg adjustment criteria for multiple comparisons miRNA‐342‐3p demonstrated the greatest value of downregulation (*p* = .02, *U* test). The cycle relative threshold (CRT) of miRNA‐342‐3p was 28.297 [27.943; 29.386] in the PC group and 27.012 [26.199; 27.309] in the PA group (Figure 2).

### 3.3 Diagnostic value of miRNA‐342‐3p

**FIGURE 3:**
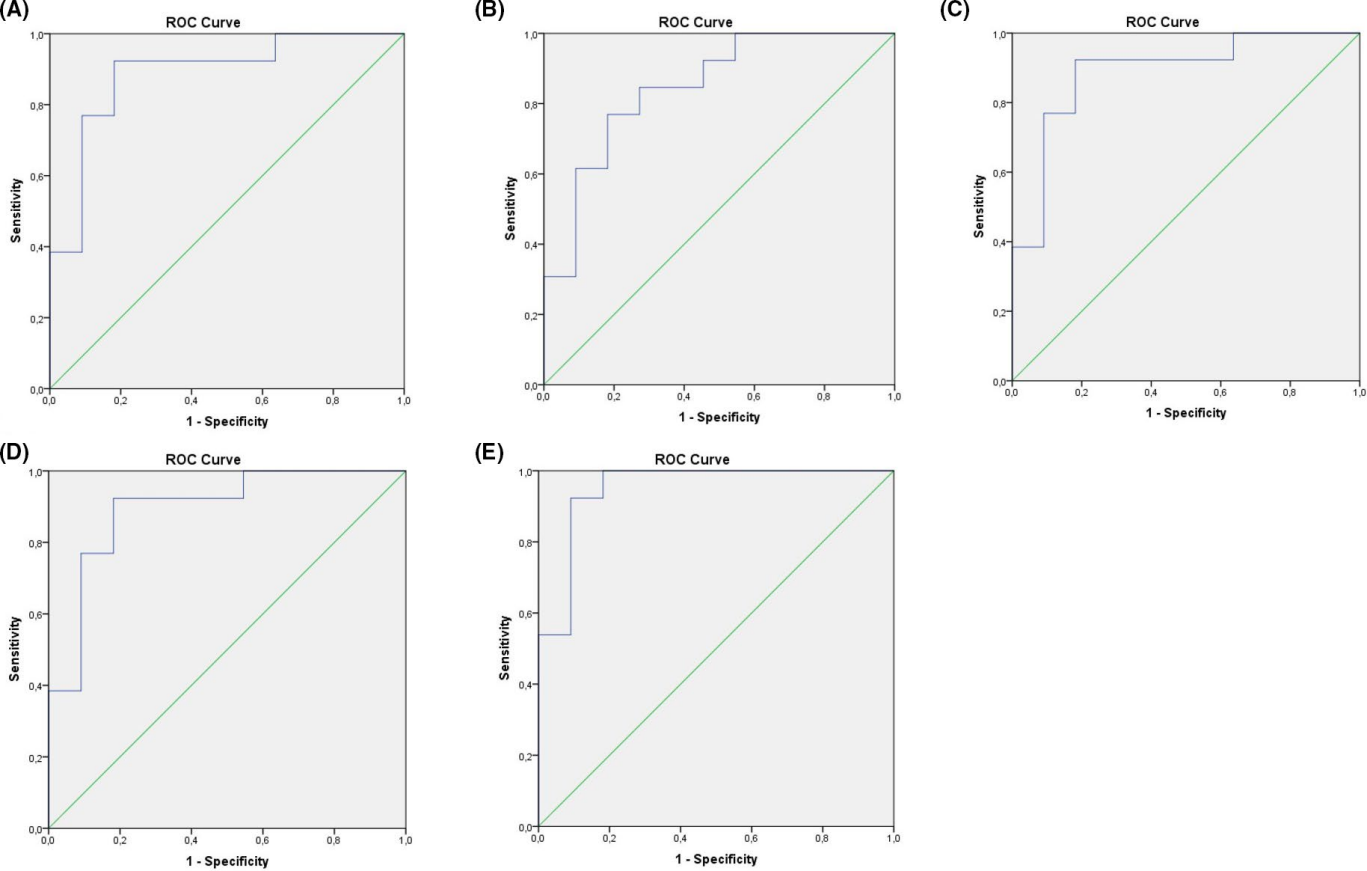
(A) Diagnostic efficacy of miRNA‐342p determined by receiver operator characteristic curve analysis (ROC). Area under curve (AUC) =0.888 (95% CI, 0.749–1; *p* = .001), which indicated the average diagnostic efficiency. (B) Diagnostic efficacy of iPTH determined by ROC curve analysis. AUC =0.846 (95% CI, 0.688–1; *p* = .004), which indicated the average diagnostic efficiency. (C) Diagnostic efficacy of calcium determined by ROC. AUC =0.846 (95% CI, 0.684–1; *p* = .004), which indicated the average diagnostic efficiency. D. Diagnostic efficacy of combination calcium and iPTH serum levels determined by ROC. AUC =0.895 (95% CI, 0.762–1; *p *= .001), which indicated the average diagnostic efficiency. E. Diagnostic efficacy of combination the CRT of the miRNA‐342p with calcium and iPTH serum levels determined by ROC. AUC = 0.951 (95% CI, 0.863–1; *p* < .001), which indicated the average diagnostic efficiency

Receiver operator characteristic (ROC) curve analysis was used to evaluate the diagnostic accuracy of the serum miRNA‐342‐3p level for the PC group. The ROC curve was constructed for the expression of miRNA‐342‐3p (CRT). The area under the curve (AUC) of the serum miRNA‐342‐3p was 0.888 (95% CI, 0.749–1; *p *= .001, Figure 3A) which indicates the average diagnostic efficiency. The cut‐off point of the miRNA‐342‐3p is 27.5 (specificity = 92%, sensitivity = 81%). Also, ROC curve analysis was used to evaluate the diagnostic accuracy of the serum PTH and calcium levels for the PC. The AUC of the new ROC curve was 0.846 (95% CI, 0.688–1; *p* = .004; Figure 3B) which indicates the average diagnostic efficiency. The corresponding cut‐off point of PTH is 531.5 pg/ml (specificity =77%, sensitivity =82%). The AUC of the serum calcium was 0.846 (95% CI, 0.684–1; *p* = .004; Figure 3C) which indicates the average diagnostic efficiency. The cut‐off point of calcium is 3.39 mmol/l (specificity = 62%, sensitivity = 100%). The ROC curve of the combination Ca and iPTH serum levels showed that the AUC was 0.985 (95% CI, 0.762–1; *p* = .001; Figure 3D). The ROC curve of the combination CRT of the miRNA‐342‐3p and serum calcium and iPTH serum levels showed that the AUC was 0.951 (95% CI, 0.863–1; *p* < .001; Figure 3E). The ROC curves do not differ statistically significantly, because confidence intervals are crossed. However, ROC curve of the combination CRT of the miRNA‐342‐3p and serum calcium and iPTH serum levels is a better result because its AUC has a more narrow confidence interval than the AUC of the only CRT of the miRNA‐342p.

Combination CRT of the miRNA‐342‐3p and serum calcium and iPTH serum levels has sensitivity = 92% and specificity = 91%.

The PC patients were divided into two subgroups [patients with (*n* = 8) and without metastases (*n* = 5)]. The analysis between subgroups did not reveal any significant difference in the CRT of the miRNA‐342‐3p (*p* = .34, *U* test). 5 out of 8 carcinomas metastasized to the lung. The analysis between the patients (*n* = 5) with metastasis to the lungs and the patients (*n* = 3) with metastasis to another organs did not reveal significant difference in the CRT of the miRNA‐342‐3p (*p* = 0.57, *U* test). There is no difference in the CRT of miRNA‐342‐3p between patients with ( n = 3) and without *CDC73* mutations (*n* = 10) (*p* = .57, *U* test). Also, analysis between patients with (*n* = 5) and without vascular invasion (*n* = 8) showed no difference in the CRT of miRNA‐342‐3p (*p* = .35, *U* test).

### 3.4 Correlations between miRNA‐342‐3p and clinical data

**FIGURE 4:**
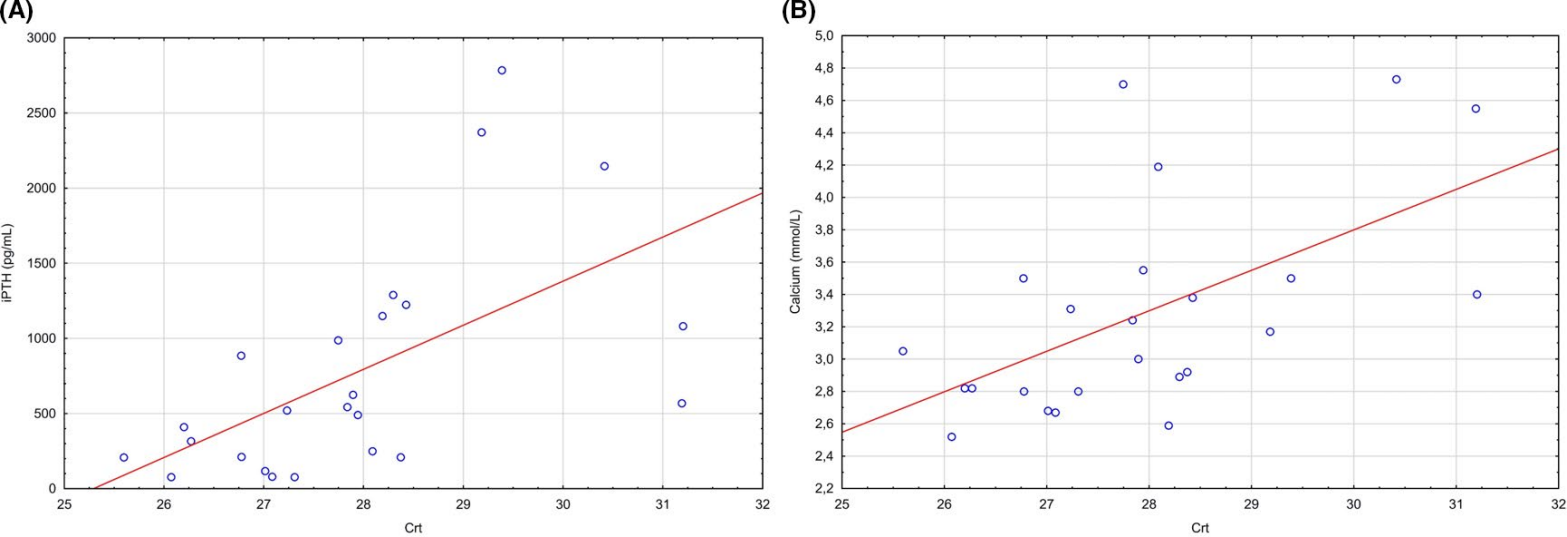
(A) Spearman's correlation analysis of calcium levels and relative miRNA‐342‐3p expression in human serum samples (*n* = 24). (B) Spearman's correlation analysis of intact parathyroid hormone (iPTH) levels and relative miRNA‐342‐3p expression in human serum samples (*n* = 24)

Associations between the serum miRNA‐342‐3p level and clinicopathological parameters were determined using Spearman's correlation analysis, which showed an average positive correlation between preoperative calcium with miRNA‐342‐3p (r_s_ = .52, *p* = .01) (Figure 4A) and preoperative iPTH, as determined by the miRNA‐342‐3p CRT (r_s_ = .68, *p* < .01) (Figure 4B).

## 4 DISCUSSION

In previous studies, miRNA signatures were examined in parathyroid tissues. For instance, miRNA was analysed in the PC tissue samples compared with the normal parathyroid glands^19^, ^20^, ^21^ and benign parathyroid tumours^20^, ^21^ that revealed significant miRNA downregulation in the PC versus normal parathyroid glands. Among these samples, the most consistently downregulated miRNAs were miRNA‐296‐5p, miRNA‐139‐3p, miRNA‐126‐5p, miRNA‐26b and miRNA‐30b^19^, ^20^; miRNA‐222, miRNA‐503 and miRNA‐517c were upregulated.^19^, ^20^, ^21^ The inhibition of the miRNA‐296‐5p expression was detected in distant metastases, while the miRNAs C19MC and miRNA‐372 were activated in the samples.^21^ Vaira and colleagues, unlike previous researchers, focused only on two miRNA clusters on chromosome 19 in a series of normal parathyroid glands, adenomas, carcinomas and distant metastatic lesions. They showed that C19MC miRNA aberrant expression was significantly enriched in the PC and accurately distinguished carcinomas from the PA. Moreover, the matched metastasis and primitive PC showed similar miRNA expression patterns.

Recently, circular RNA profiles were described in the PC for the first time, suggesting that those also play a role in parathyroid tumorigenesis.^26^ A number of molecular mechanisms may deregulate miRNA expression in the PCs. The loss of parafibromin, which interacts with the RNA polymerase II (Pol II),^27^ may potentially alter the miRNA expression due to its role as a key molecule in the miRNA transcription. The relationship between the loss of parafibromin in the PCs and the miRNA expression has not been investigated yet.

Our results showed significant downregulation of the miRNA‐342‐3p expression in the PC group. In previous studies of the miRNA in the parathyroid tissue, the differences in the miRNA‐342‐3p expression were not found. The poor reproducibility of the results on the miRNA in parathyroid glands’ disorders between available studies could be related to different types of normal controls (or to the absence of control at all) as well as to different analysis platforms used. The results would need to be tested on additional independent cohorts to provide evidence of potential utility in further research. Among the other 16 miRNAs in our study, downregulation of the miRNA‐126 (*p *= .039, before multiple comparisons) was overlap with results of Reza Rahbari's study at all. In that work of three of the 13 selected miRNAs were significantly downregulated between adenoma and carcinoma (miR‐126, miR‐26b and miR‐30b; *p* < 0.05). The miRNA with the highest accuracy was miR‐126* (AUC = 0.766).^20^ Various studies have demonstrated that the miRNA‐342‐3p is crucial for numerous physiological and pathological processes and negatively regulates the cell viability by repressing the anti‐apoptotic gene network in human and mouse macrophages,^28^ by enhancing adipogenesis^29^ and by playing a role in the osteogenic differentiation of umbilical cord mesenchymal stem cells.^30^ *In vitro* and *in vivo* studies have shown that exogenous overexpression of the miRNA‐342‐3p in colorectal, cervical, prostate, breast, hepatocellular and non‐small‐cell lung cancer (NSCLC) induced apoptosis and inhibited the tumorigenicity, cell growth, invasion and migration through the establishment of diverse cancer regulatory networks, suggesting potential tumour suppressor activity and sensitivity of malignant cells to anticancer medicines.^31^ It has also been reported that the miRNA‐342‐3p can be a tumour suppressor miRNA in different malignancies, including hepatocellular carcinoma, cervical cancer and lung cancer. However, some results demonstrated that its suppression was associated with poor cancer prognosis (Supplementary Material). However, at present, the role of miRNA‐342‐3p in the PC is still not well understood. Interestingly, most of the deregulated miRNAs in the PC were repressed, including the revealed miRNA342‐3p in this study, suggesting onco‐suppressive roles in this disease.

Interestingly, noncoding RNAs (ncRNAs) could be also applied as potential therapeutics in the near future. The replacement of the tumour suppressor miRNA‐34a by using MRX34, the synthetic product, exhibited promising initial results in the first phase I study in humans.^32^ Since then, the usage of ncRNAs in cancer therapy is permanently improving. The data are available suggesting that ncRNAs could be implicated in predicting the chemo‐ and radio‐resistance, as well as the therapeutic targets.^33^ Interactions between the miRNAs and Toll‐like receptors have been reported underlining the implication of ncRNAs in the cancer immunology.^34^ miRNA expression patterns were also analysed to better understand and direct the immune checkpoint inhibitor therapy.^35^

The purpose of this study is not to compare the value of calcium, PTH and miRNA with each other, but to find a new marker or model for PC generally and to confirm the hypothesis that the circulating miRNA can act as a preoperative marker to distinguish the PC from the PA. Currently, the cut‐off points of calcium and iPTH for the differential diagnosis of malignant and benign parathyroid neoplasms have not been determined. More specific markers of PC are required for preoperative differential diagnosis since PA could be accompanied by severe course of PHPT with severe hypercalcemia and a significant increase in PTH, and vice versa, PC with a mild course and even normocalcaemia. A logistic regression models seem to be more reliable with the inclusion of several clinical important parameters for preoperative diagnostics (including serum calcium, PTH, but not limited to them).^36^, ^37^ In the present pilot study, circulating miRNA was proposed as a potential surrogate marker of the PC. Indeed, the AUCs of miRNA‐342‐3p, serum calcium and PTH levels are equivalent; in the same time, the combination of them appears to be the most promising preoperative models to distinguish the PC from the PA in individuals with increased risk of malignant tumour. Thus, the miRNA‐342‐3p is significantly different and a further 16 miRNAs demonstrated a p‐value of <0.05 and thus may become of interest in a larger study.

### 4.1 Benefits and limitations of the study

Our study is hypothesis setting, and thus, it will require further validation including the assessment of the circulating miR‐342‐3p level variations in the healthy control group as well as intra‐individual, circadian and food intake variations. The sample size in the study is relatively small, as the PC is an extremely rare disease and patients in most cases had undergone multiple interventions. However, we had recruited a cohort of 13 PC patients on the diagnosis phase and before the surgical intervention. Thus, we aimed to obtain the pilot data to understand whether there are promising differences, which can be used in further biomarker research and should be confirmed in tissue samples. However, we must consider several limitations of the circulating miRNAs as the biomarkers in cancer patients. First, the miRNA expression levels cannot be used as biomarkers of specific cancers, because dysregulation of the same miRNA can be observed in different types of cancer and diseases (Supplementary Material). In addition, the differences between the two groups could be related to other, non–cancer‐related, differences, for example height of Ca or iPTH levels. Second, some miRNAs may have opposite expression patterns depending on the cancer type. Thus, the miRNA could be an additional tool for the preoperative diagnosis of the PC and to identify the groups with increased cancer risk (high serum Ca and iPTH levels). Despite these limitations, this approach has several benefits. All the included patients were clinically well‐characterized. All the samples were collected at the same time period using the same collection technique and the identical lot number of blood‐collection tubes. The exact reagent set was used for all analysed samples. Moreover, differences in the miRNA‐342‐3p expression have remained significant after Benjamini‐Hochberg adjustments, which is a promising result for further investigation.

## 5 CONCLUSION

The main objective of this study was to compare the serum miRNA expression profiles between the patients with benign and malignant parathyroid tumours. Our results demonstrated significant downregulation of the miRNA‐342‐3p expression in the PC group. In this way, the serum miRNA‐342‐3p levels may serve as a potential preoperative biomarker of the PC in patients with high risk for the malignant tumour, as well as postoperative monitoring of the disease coupled with the iPTH and calcium levels. This work motivates further investigations required to prove the efficiency and reliability of the proposed biomarkers.

## CONFLICTS OF INTEREST

The article is part of the Ph.D. work of Julia Krupinova. The authors declare no conflict of interest.

## AUTHOR CONTRIBUTIONS

J.K., A.E., E.P. and N.M designed the project and supervised the experiments; J.K. and V.P. performed the experiments and data analysis; A.T. validated the study, J.K., E.P. and V.P. wrote—original draft preparation; N.M, A.E., E.D. and G.M. wrote—review and editing; A.A. performed statistical analysis. All authors have read and agreed to the published version of the manuscript.

## Supporting information

Supplementary Material

Supplementary Material

## Data Availability

The data that support the findings of this study are available from the corresponding author upon request.

## References

[1] Hundahl SA , Fleming ID , Fremgen AM , Menck HR . Two Hundred Eighty‐six cases of parathyroid carcinoma treated in the U.S. Between. Cancer. 1999;86(3):538‐544. 10.1002/(sici)1097-0142(19990801)86:3<538:aid-cncr25>3.0.co;2-k 10430265

[2] Lee PK , Jarosek SL , Virnig BA , Evasovich M , Tuttle TM . Trends in the incidence and treatment of parathyroid cancer in the United States. Cancer. 2007;109(9):1736‐1741. 10.1002/cncr.22599 17372919

[3] Brown S , O’Neill C , Suliburk J , et al. Parathyroid carcinoma: increasing incidence and changing presentation. ANZ J Surg. 2011;81(7–8):528‐532. 22295377 10.1111/j.1445-2197.2010.05594.x

[4] Kebebew E , Arici C , Duh QY , Clark OH . Localization and reoperation results for persistent and recurrent parathyroid carcinoma. Arch. Surg. Chic. Ill. 2001;136(8):878‐885. doi:10.1001/archsurg.136.8.878 11485522

[5] Asare EA , Sturgeon C , Winchester DJ , et al. Parathyroid carcinoma: an update on treatment outcomes and prognostic factors from the National Cancer Data Base (NCDB). Ann Surg Oncol. 2015;22(12):3990‐3995. 10.1245/s10434-015-4672-3 26077914

[6] Lo WM , Good ML , Nilubol N , Perrier ND , Patel DT . Tumor size and presence of metastatic disease at diagnosis are associated with disease‐specific survival in parathyroid carcinoma. Ann Surg Oncol. 2018;25(9):2535‐2540. 10.1245/s10434-018-6559-6 29971678 PMC8054302

[7] Wei CH , Harari A . Parathyroid carcinoma: update and guidelines for management. Curr Treat Options Oncol. 2012;13(1):11‐23. 10.1007/s11864-011-0171-3 22327883

[8] Mokrysheva N , Mirnaya S , Dobreva E , et al. Primary hyperparathyroidism in Russia according to the registry. Problems of Endocrinology. 2019;65(5):300‐310. 10.14341/probl10126 32202734

[9] Newey PJ . Cell division cycle protein 73 homolog (CDC73) mutations in the hyperparathyroidism‐jaw tumor syndrome (HPT‐JT) and parathyroid tumors. Hum Mutat. 2010;31(3):295‐307. 10.1002/humu.21188 20052758

[10] Villar‐del‐Moral J , Jimenez‐Garcia A , Salvador‐Egea P , et al. Prognostic factors and staging systems in parathyroid cancer: a multicenter cohort study. Surgery. 2014;156(5):1132‐1144. 10.1016/j.surg.2014.05.014 25444314

[11] Bollerslev J , Schalin‐Jantti C , Rejnmark L , et al. PARAT Workshop Group. Management of endocrine disease: unmet therapeutic, educational and scientific needs in parathyroid disorders. Eur J Endocrinol. 2019;181(3):1‐19. 10.1530/EJE-19-0316 31176307 PMC6598862

[12] Schulte KM , Talat N , Galata G , et al. Oncologic resection achieving R0 margins improves disease‐free survival in parathyroid cancer. Ann Surg Oncol. 2014;21(6):1891‐1897. 10.1245/s10434-014-3530-z 24522991

[13] Spinelli C , Bonadio AG , Berti P , Materazzi G , Miccoli P . Cutaneous spreading of parathyroid carcinoma after fine needle aspiration cytology. J Endocrinol Invest. 2000;23(4):255‐257. 10.1007/BF03343718 10853713

[14] Kozomara A , Griffiths‐Jones S . MiRBase: annotating high confidence microRNAs using deep sequencing data. Nucleic Acids Res. 2014;42:68‐73. 10.1093/nar/gkt1181 PMC3965103 24275495

[15] Romero‐Cordoba SL , Salido‐Guadarrama I , Rodriguez‐Dorantes M , Hidalgo‐Miranda A . MiRNA biogenesis: biological impact in the development of cancer. Cancer Biol Ther. 2014;15(11):1444‐1455. 10.4161/15384047.2014.955442 25482951 PMC4622859

[16] Anfossi S , Babayan A , Pantel K , Calin GA . Clinical utility of circulating non‐coding RNAs ‐ an Update. Nat. Rev. Clin. Oncol. 2018;15(9):541‐563. 10.1038/s41571-018-0035-x 29784926

[17] Chen X , Ba Y , Ma L , et al. Characterization of microRNAs in serum: a novel class of biomarkers for diagnosis of cancer and other diseases. Cell Res. 2008;18(10):997‐1006. 10.1038/cr.2008.282 18766170

[18] Pardini B , Sabo AA , Birolo G , Calin GA . Noncoding RNAs in extracellular fluids as cancer biomarkers: the new frontier of liquid biopsies. Cancers. 2019;11(8):1170. 10.3390/cancers11081170s PMC6721601 31416190

[19] Corbetta S , Vaira V , Guarnieri V , et al. Differential expression of microRNAs in human parathyroid carcinomas compared with normal parathyroid tissue. Endocr Relat Cancer. 2010;17(1):135‐146. 10.1677/ERC-09-0134 19926710

[20] Rahbari R , Holloway AK , He M , Khanafshar E , Clark OH , Kebebew E . Identification of differentially expressed microRNA in parathyroid tumors. Ann Surg Oncol. 2011;18(4):1158‐1165. 10.1245/s10434-010-1359-7 21086055 PMC3449317

[21] Vaira V , Elli F , Forno I , et al. The microRNA cluster C19MC is deregulated in parathyroid tumours. J Mol Endocrinol. 2012;49(2):115‐124. 10.1530/JME-11-0189 22767050

[22] Hu Y , Zhang X , Cui M , et al. Verification of candidate microRNA markers for parathyroid carcinoma. Endocrine. 2018;60(2):246‐254. 10.1007/s12020-018-1551-2 29453660

[23] DeLellis RA , Arnold A , Bilezikian JP . WHO Classification of Tumours of Endocrine Organs // Parathyroid Carcinoma. 2017;147‐158.

[24] Mestdagh P , Van Vlierberghe P , De Weer A , et al. Novel and universal method for microRNA RT‐QPCR data normalization. Genome Biol. 2009;10(6):64. 10.1186/gb-2009-10-6-r64 PMC2718498 19531210

[25] BBenjamini Y , Hochberg Y . Controlling the false discovery rate: a practical and powerful approach to multiple testing. J R Stat Soc Ser B Methodol. 1995;57(1):289‐300.

[26] Hu Y , Zhang X , Cui M , et al. Circular RNA profile of parathyroid neoplasms: analysis of co‐expression networks of circular RNAs and MRNAs. RNA Biol. 2019;16(9):1228‐1236. 10.1080/15476286.2019.1622962 31213128 PMC6693544

[27] Rozenblatt‐Rosen O , Hughes CM , Nannepaga SJ , et al. The parafibromin tumor suppressor protein is part of a human Paf1 complex. Mol Cell Biol. 2005;25(2):612‐620. 10.1128/MCB.25.2.612-620.2005 15632063 PMC543415

[28] Czimmerer Z , Varga T , Kiss M , et al. The IL‐4/STAT6 signaling axis establishes a conserved microRNA signature in human and mouse macrophages regulating cell survival via MiR‐342‐3p. Genome Med. 2016;8(1):63. 10.1186/s13073-016-0315-y 27245778 PMC4886428

[29] Wang L , Xu L , Xu M , et al. Obesity‐associated MiR‐342‐3p promotes adipogenesis of mesenchymal stem cells by suppressing CtBP2 and releasing C/EBPalpha from CtBP2 binding. Cell Physiol Biochem. Int J Exp Cell Physiol Biochem Pharmacol. 2015;35(6):2285‐2298. 10.1159/000374032 25895816

[30] Huang M , Qing Y , Shi Q , Cao Y , Song K . MiR‐342‐3p elevates osteogenic differentiation of umbilical cord mesenchymal stem cells via inhibiting sufu in vitro. Biochem Biophys Res Commun. 2017;491(3):571‐577. 10.1016/j.bbrc.2017.07.163 28765042

[31] Crippa E , Lusa L , De Cecco L , et al. MiR‐342 regulates BRCA1 expression through modulation of ID4 in breast cancer. PLoS ONE. 2014;9(1):87039. 10.1371/journal.pone.0087039 PMC3903605 24475217

[32] Pichler M , Calin GA . MicroRNAs in cancer: from developmental genes in worms to their clinical application in patients. Br J Cancer. 2015;113(4):569‐573. 10.1038/bjc.2015.253 26158421 PMC4647691

[33] Wang W‐T , Han C , Sun Y‐M , Chen T‐Q , Chen Y‐Q . Noncoding RNAs in cancer therapy resistance and targeted drug development. J Hematol Oncol J Hematol Oncol. 2019;12(1):55. 10.1186/s13045-019-0748-z 31174564 PMC6556047

[34] Bayraktar R , Bertilaccio MTS , Calin GA . The interaction between two worlds: microRNAs and Toll‐like receptors. Front Immunol. 2019;10:1053. 10.3389/fimmu.2019.01053 31139186 PMC6527596

[35] Cortez MA , Anfossi S , Ramapriyan R , et al. Role of MiRNAs in immune responses and immunotherapy in cancer. Genes Chromosomes Cancer. 2019;58(4):244‐253. 10.1002/gcc.22725 30578699 PMC6368474

[36] Schulte KM , Talat N . Diagnosis and management of parathyroid cancer. Nat Rev Endocrinol. 2012;8(10):612‐622. 10.1038/nrendo.2012.102 22751344

[37] Liu R , Xia Y , Chen C , et al. Ultrasound combined with biochemical parameters can predict parathyroid carcinoma in patients with primary hyperparathyroidism. Endocrine. 2019;66(3):673‐681. 10.1007/s12020-019-02069-7 31489590

