# Supplementary material for: Serum circulating miRNA‐342‐3p as a potential diagnostic biomarker in parathyroid carcinomas: A pilot study
Source: Endocrinol Diabetes Metab. 2021 Jul 29;4(4):e00284. doi: 10.1002/edm2.284 (PMC8502227; doi:10.1002/edm2.284)
Supplement: Supplementary file 1 — Supplementary Material [file EDM2-4-e00284-s002.docx]

**Appendices.**

The roles of miRNA-342-3p in human cancer.

| **Lead author, year** | **Pathology/ cancer** | **Substrate** | **Expression** | **Prognostic or clinical value** |
| --- | --- | --- | --- | --- |
| Gao Y., 2017 | Hepatocellular carcinoma | Tissues | Downregulation | Correlation with poor survival rate |
| Li X-R., 2014 | Cervical cancer | The human cervical cancer cell lines | Downregulation | Negative correlation with transcription factor FOXM1, which is responsible for the grade of cervical intraepithelial neoplasia and cervical cancer |
| Xie X., 2015 | Non-small cell lung cancer (NSCLC) | NSCLC tissues and cell lines and | Downregulation | Raise of NSCLC cell proliferation, migration, and invasion |
| Qin Y., 2018 | NSCLC | Cell lines and serum samples | Downregulation | Correlation with worse overall survival |
| Xue X., 2018 | NSCLC | NSCLC tissues and cell lines | Downregulation | Negative correlation with anterior gradient protein 2 expression, which is associated with worse prognosis |
| Wang C., 2019 | Оvarian carcinoma | Tissues and cell lines | Downregulation | Negative correlation with FOXQ as an oncogene in ovarian cancer |
| [Zhang W](https://www.ncbi.nlm.nih.gov/pubmed/?term=Zhang%20W%5BAuthor%5D&cauthor=true&cauthor_uid=28112756)., 2017 | Glioma | Tissues and the human glioma cell lines | Downregulation | Association with overexpression of long noncoding RNA FTX and glioma progression |
| Cui Z., 2019 | Nasopharyngeal carcinoma | Tissues and cell lines | Downregulation | Correlation with reduced overall survival |
| Song X., 2019 | Oral squamous cell carcinoma (OSCC) | Tissues and human oral squamous cell lines | Downregulation | Promotion of the OSCC cells proliferation |
| Romero-Cordoba SL., 2018 | Triple negative breast cancer | Human breast cancer tissues and cell lines | Downregulation | Association with increased monocarboxylate transporter (MCT1) expression and glycolytic score in human triple-negative tumors |
| Zhang S., 2017 | Osteosarcoma | Osteosarcoma tissues and cell lines | Downregulation | Increased proliferation, migration, and invasion of osteosarcoma cells through AEG-1 gene targeting |
| Wang SH. 2016 | Gallbladder cancer | Tissues | Downregulation | Association with long noncoding RNA (lncRNA) H19 as an oncogene |
| De Marchis ML. 2009 | Leukemia | Cell lines | Downregulation | Association with all-trans-retinoic acid (ATRA) treatment |
| Dacic S., 2010 | Lung adenocarcinoma | Tissues | Downregulation | - |
| Fayyad-Kazan H., 2013 | Acute myeloblastic  leukemia | Plasma samples | Downregulation | Normal expression in patients in remission |
| Cheng D., 2019 | Pancreatic cancer | Tissues and cell lines | Downregulation | Association with small nuclear RNA host gene 7  (SNHG7) overexpression, which is associated with poor prognosis |
