# Supplementary material for: Serum circulating miRNA‐342‐3p as a potential diagnostic biomarker in parathyroid carcinomas: A pilot study
Source: Endocrinol Diabetes Metab. 2021 Jul 29;4(4):e00284. doi: 10.1002/edm2.284 (PMC8502227; doi:10.1002/edm2.284)
Supplement: Supplementary file 2 — Supplementary Material [file EDM2-4-e00284-s001.docx]

Here is the drop box link for the appendix 2b, which can not be uploaded onto JCN website due to file size limit: "https://www.dropbox.com/sh/mol8s92td4gnirp/AAAEezL-OZvMXc2trRobHZjWa?dl=0"
